# Developing and Assessing the Validity of a Scale to Assess Pet Dog Quality of Life: Lincoln P-QoL

**DOI:** 10.3389/fvets.2019.00326

**Published:** 2019-09-26

**Authors:** Sophie S. Hall, Beverley J. Brown, Daniel S. Mills

**Affiliations:** ^1^Animal Behaviour Cognition and Welfare Group, School of Life Sciences, University of Lincoln, Lincoln, United Kingdom; ^2^Division of Psychiatry and Applied Psychology, School of Medicine, University of Nottingham, Nottingham, United Kingdom

**Keywords:** yawn, calming signal, behavior, child-dog interactions, scale development, quality of life, pet dog

## Abstract

There has been little investment in exploring the impact of the child-dog relationship on the dog. Since child-dog interactions can pose potentially serious threats to a dog's physical and psychological health, as well as the wider satisfaction of the owner with their dog, we describe the development and validation of an owner-completed pet dog quality of life scale (Lincoln P-QOL), to enable professionals and families to monitor dog well-being and employ suitable interventions as required. Four-hundred and two dog-owners (194 lived with a neuro-typically developing child; 208 lived with a child with a neuro-developmental disorder) responded to an online survey. Respondents recorded whether they had observed their dog displaying any of the 22 behavioral responses which have been identified as being common in 11 child-dog interactions. These behavioral responses appeared to group into three categories of behaviors (i.e., behavioral constructs), representing Excitability, Calmness, and Fearfulness in the dog. To assess convergent validity of the quality of life scale respondents completed additional measures including, dog body condition score, health issues (incorporating psychological factors such as anxiety and physical proxies of well-being, such as skin irritations) and dog-owner relationship satisfaction. Excitability and Fearfulness constructs were associated with a negative impact on dog health and the owner-dog relationship. Calmness was associated with a positive impact on the dog-owner relationship. A range of interactions, including carefully expressed child-dog physical affection and spending quiet time together appear to had a beneficial impact on dog quality of life, whereas rough contact, child meltdowns, and grooming/bathing had a negative effect. We found little evidence to support a difference in the overall quality of life of dogs living with neuro-typically developing children compared to those with a neuro-developmental disorder. However, parents and practitioners need to be aware of the potential increased risk to dog well-being when meltdowns, grooming/bathing, and quiet time involve a child with a neuro-developmental disorder. This is the first validated scale for the assessment of dog well-being around children, additionally, the behavioral constructs identified may form the rational basis of a more general dog behavior/stress assessment tool in social situations.

## Introduction

There is growing awareness and interest in the value that pet dogs, with no formal training, can bring to human health and well-being, especially in relation to child development (1–4). In particular, companionship associated with pet dog ownership may benefit children, and their families, affected by neuro-developmental disorders such as Autism Spectrum Disorder (ASD) (5–11). Reports of these benefits may persuade families to acquire a pet dog with unrealistic expectations of the owner-dog relationship (12, 13), and without informed consideration of the impact on the dog. Here, we explore this impact using the term “quality of life,” since this is a multi-dimensional concept which encapsulates satisfaction across a number of domains (e.g., psychological, physical, and social). Given our focus on family dogs this may be a preferable point of reference than the dog's physical well-being alone when examining the effect of risk factors on these dogs (14). However, the general literatures typically focus on dog “well-being” (i.e., physical impacts/behavioral symptoms of stress). Indeed, although a range of disease specific quality of life instruments have been developed [e.g., (15–18)], relatively little attention has been paid to dog general quality of life (19).

Dog behaviors related to stress, fear and anxiety are those most likely to be perceived as problematic by their owners (20). Regular displays of problematic behaviors in dogs are associated with breakdown in the owner-dog relationship (21, 22). Relationship breakdown is the leading cause of behavior problems and relinquishment of dogs to shelters (23, 24). Therefore, ignoring the potential implications of living with children on dog well-being may negatively affect dog quality of life (through changes in both physical and psychological health), and satisfaction with the owner-dog relationship (25, 26), as well as increasing the risk of child-directed aggression. Indeed, fear and anxiety, along with environmental and social stressors likely to cause frustration are associated with irritability and aggressive displays in dogs (27–29). For instance, although children often love to be physically close to, and tactile with a dog (30), dogs do not always share the enjoyment of this contact, as evidenced by their body language, and it is these types of child-initiated interactions which can often lead to dog bites (31, 32). Physiological studies suggest that even with adults, some dogs may find certain close contact interactions (e.g., kissing, petting) stressful, as evidenced by increases in cortisol (33, 34). Nonetheless, some studies also suggest that some dogs may demonstrate an increase in the positive social-bonding hormone, oxytocin, the more the owner kisses the dog (35). Thus, it is unwise to make simple generalizations concerning how close social interactions may be perceived by dogs in general.

Somewhat surprisingly, little research has explored the relationship between child-dog interactions and dog quality of life; indeed a recent systematic review in this area (14) highlighted that only five published articles have reported data on the impact of child-dog interactions for dog quality of life (36–40). The review by Hall et al. (14) highlighted that evidence comes from diverse measures including altered behaviors, physical well-being and social interactions. Potentially problematic child-dog interactions identified in the review paper could be broadly grouped into three categories: unprovoked attention (e.g., aggressive child behaviors associated with meltdowns, cuddling, and kissing), environmental predictability (e.g., lack of peace time, routine), and child games (e.g., fancy dress). The review also highlights that existing research exploring the impact of child-dog interactions on the dog focuses almost exclusively on trained assistance dogs (14). However, pet dogs do not typically receive much, if any, training or preparation for interacting with children, therefore, they may be less resilient in the interactions and so at greater risk. Indeed, only one study explored the impact of living with neuro-typically developing children and children with a neuro-developmental disorder on pet dog quality of life (38). Based on qualitative parent interviews this study identified 11 child-dog interactions which could be a potential source of stress for pet dogs including: child meltdowns, child and dog being in the car together, child visitors at the home, child cuddles and/or kisses the dog, child grooms and/or bathes the dog, child engages in high energy activities with the dog, child plays with wheeled and/or loud toys, child disrupts the dogs routine for one reason or another, child is rough with the dog, child and dog sit quietly together, child disturbs their dog. The qualitative descriptions of theses interactions did not differ much between families with neuro-typically developing children and children with a neuro-developmental disorder, and so may impact similarly on the dog regardless of the child's developmental status. However, quantitative comparisons between dogs living with these two groups of children have yet to be investigated, additionally, behavioral impact of child-dog interactions have not been explored. In the interests of dog well-being, it is essential that we develop methods to assess the impact of child-dog interactions (involving typically developing children and those with a neuro-developmental disorder) on the dog, so that professionals and families can monitor dog well-being in known-risk situations and employ suitable interventions as required. Any method must be feasible, easy to implement and validated (41). Therefore, the aim of this study was to explore the child-dog relationship from the dog's perspective, through the development and initial validation of an owner completed scale to assess dog quality of life, specifically focused on the risks posed by child-dog interactions. We also sought to explore predictors of dog quality of life when living with children, and to compare the impact of living with children with a neuro-developmental disorder and neuro-typically developing children, on the dog It was hypothesized that quality of life may be evaluated from the identification of behaviors associated with positive and negative states of arousal. It was further predicted that positive effects of child-dog interactions on the dog would be associated with positive outcomes on other measures of well-being (e.g., body condition score, health issues, owner-relationship satisfaction). Additionally, it was expected that if child-dog interactions were experienced differently by the dog if they involved a child with a neuro-developmental disorder compared to a neuro-typically developing child, then this would be reflected in quality of life scores.

## Method

### Participants

The study received ethical approval from the University of Lincoln's College of Science Research Ethics Committee (ID: CoSREC355). Participants were recruited via press releases, social media and through the University of Lincoln's database of dog owners. Study advertisements stated that we were interested in exploring the child-dog relationship and we were looking for dog-owning parents of children (aged 3–16 years) to take part in a short survey. Advertisements specified that we were keen to hear from families who have typically developing children and those with neuro-developmental disorders (e.g., autism, attention deficit hyperactivity disorder). A prize draw for a £50 voucher was offered as an incentive to complete the survey. Interested participants were directed to the study website.

### Website

Twenty-two behaviors related to the immediate assessment (i.e., as currently presented) of dog well-being were selected based on previous research (26, 38, 42). A project website was set-up to help parents identify these behaviors. The dog behaviors listed were: repetitive behaviors (such as tail chasing, circling, or pacing), chewing objects, listless or withdrawn, yawning, bouncing/jumping up, panting (unrelated to exercise), cowering (low body posture), aggression toward the child (including growling, snapping), hiding, seeking safety or comfort, vocalizing (e.g., barking, whining), lip or nose licking, wide eyes or worried eyes, shaking/trembling, ignoring physical or verbal commands, running away, moving closer to the child, play bow/play behavior without an object, laying down/relaxed, playing with an object/toy, high tail wag, and responding positively to verbal or physical commands. For each behavior a short description and a short video was provided. Although it could be argued that some of these behaviors should be separated (e.g., distinct items for tail chasing and circling, or grooming and bathing) our qualitative work in this area indicates, that at least at this stage of enquiry in this field, these behaviors can be combined without losing meaning (38). Furthermore, their relevance (face validity as measures of well-being) and the clarity of descriptions were confirmed by an expert panel (comprised of veterinary behaviorists, pet dog support charity workers, and dog owning parents), utilizing their clinical knowledge and/or experience. Respondents were advised to use the visual and descriptive information to help them recognize specific dog behaviors, but it was also mentioned that this information should be used as a guide only, since different dogs may display the behaviors in slightly different ways (e.g., the way a Labrador wags its tail is different to the way a pug does it). Respondents were asked if they found the website useful to help them identify dog behaviors. The project website also contained information about the study team as well as links to useful contacts (e.g., support charities and accredited animal behaviorists).

### Survey

The survey was hosted using Qualtrics, a secure online server. In the introduction, respondents were informed about the purpose of the survey and told there were no right or wrong answers. Links were provided to the study website. Respondents were required to confirm that they had viewed the website information before continuing with the survey. They were also asked to confirm that they: currently owned a pet dog, which they had owned for over 1 year; they lived with a child aged 3–16 years; that they were happy to answer the survey questions with one dog and one child in mind (for multiple child and dog-owning families); that they were aged 18 years or over. Respondents were advised the survey would take ~20–25 min to complete.

Participants were asked a number of demographic questions (see Table 1), including: child age, dog age, length of time dog owned, relationship to the child, whether they were the main caregiver to the child and the dog, dog pedigree status, dog weight category, dog training status (responses were subsequently re-categorized, see later), whether the child was neuro-typically developing or had a clinically confirmed neuro-developmental disorder and what the child's primary diagnosis was.

**Table 1:** Sample characteristics.

	**Neuro-typically developing (*n* = 194)**	**Neuro-developmental disorder (*n* = 208)**
	**Mean** **±** **SD**	
Child age	8.3 years ± 3.9	10.4 years ± 3.4
Dog age	5.2 years ± 3.3	4.9 years ± 3.2
Time dog owned	4.7 years ± 3.2	4.3 years ± 3.03
	**% (*****n*****)**	
**Respondents relationship to child**		
Mother	92.3% (179)	93.3% (194)
Father	3.1% (6)	1.4% (3)
Non-biological female parent	2.6% (5)	2% (4)
Non-biological male parent	0% (0)	0.5% (1)
Sister	0% (0)	1% (2)
Brother	0% (0)	0% (0)
Grandmother	1% (1)	1% (2)
Grandfather	0% (0)	0% (0)
Auntie	1% (1)	1% (2)
Uncle	0% (0)	0% (0)
**Respondent main caregiver to**		
Child (yes)	96.9% (188)	96.2% (200)
Dog (yes)	97.9% (190)	95.% (199)
**Dog breed**		
Single	55.2% (107)	52.4% (109)
Mixed	44.8% (87)	47.6% (99)
**Dog weight**		
<5 kg	4.1% (8)	6.3% (13)
5–20 kg	54.6% (106)	56.3% (117)
>20 kg	41.2% (80)	37.5% (78)
**Dog training**		
Obedience	39.2% (76)	40.9% (85)
Assistance dog	0.5 (1)	1.9% (4)
Family dog	0% (0)	1.4% (3)
Kennel Club (KC)	2.6% (5)	2.9% (6)
Working dog	1% (2)	1.4% (3)
Scent work	2.6% (5)	1% (2)
Agility	1% (2)	1% (2)
Obedience and agility	6.7% (13)	5.8% (12)
Working and scent	2.1% (4)	1.4% (3)
KC and agility	1% (2)	1% (2)
Scent and agility	0% (0)	1% (2)
Assistance dog and agility	0.5% (1)	0% (0)
KC and working	1% (2)	0.5% (1)
KC and scent	0.5% (1)	0.5% (1)
Family dog and agility	0% (0)	0.5% (1)
Working and agility	0.5% (1)	0.5% (1)
No specific	39.2% (76)	35.6% (74)
Other	1.5% (3)	2.9% (6)
**Child's primary diagnosis**		
Autism spectrum disorder	-	74% (154)
Attention-deficit hyperactivity disorder	-	13.9% (29)
Tourette's syndrome	-	0.5% (1)
Intellectual disability	-	0.5% (1)
Communication disorder	-	1.4% (3)
Motor disorder	-	1% (2)
Specific learning disorder	-	1.4% (3)
Decline to answer	-	1.9% (4)
Other	-	5.3% (11)
**MEASURES OF WELL-BEING AND RELATIONSHIP SATISFACTION**		
**Body score**		
1	1% (2)	1.9% (4)
2	0% (0)	0.5% (1)
3	7.7% (15)	6.3% (13)
4	27.8% (54)	20.7% (43)
5	38.7% (75)	46.6% (97)
6	22.7% (44)	20.7% (43)
7	2.1% (4)	2.9% (6)
8	0% (0)	0.5% (1)
9	0% (0)	0% (0)
	**Mean** **±** **SD**	
Total health issues	2.1 ± 1.8	2.1 ±1.9
Relationship satisfaction	31.8 ± 3.3	31.8 ± 3.3

The second part of the survey pertained to measures of well-being and relationship satisfaction. Respondents were asked to rate their dog's body condition score, using a standard 9-point scale (43) as used in previous research (42). They were also asked 14 questions regarding their dog's general health (physical and psychological proxies) in the context of a stressful environment. These proxies of health were based on relevant literature [e.g., (26)] and in consultation with veterinary behavioral experts, who deemed these questions to be sufficiently comprehensive to provide a brief proxy of dog well-being Using a binary 1/0 scoring system to promote objective responses (42), respondents were asked if their dog tended to suffer from skin scurf/flakes, mucky eyes, regular bladder problems, sexual/reproductive issues (false pregnancies, inappropriate mounting), allergies/itchiness, digestive/tummy issues, anxious when left alone—or at the thought of being left (e.g., displays excessive vocal behaviors, are destructive, and/or toilet in the house), a generally anxious temperament, was scared by certain sounds (e.g., fireworks), nervous of new places, disliked certain people (excluding people they have had a bad experience with, such as the vet), was overly reactive—but without showing signs of being scared (e.g., barks a lot), licked or scratched itself a lot, appeared stiff/had an unusual gate.

In the absence of an established questionnaire to assess owner relationship satisfaction with their dog we adapted a validated, widely-applied human scale which provides a generic measure of relationship satisfaction, the Relationship Assessment Scale (RAS) (44). The 7-items (meet needs, satisfied with relationship, how good is relationship compared to other owners, wish hadn't got dog, meet original expectations, love dog, problems in relationship) which comprise this unifactorial scale provide a general assessment of relationship satisfaction making them suitable for adaptation for use in this study, for instance “how well does your partner meet your needs” was altered to read “how well does your dog meet your needs.” Alpha coefficients for the adapted scale (α = 0.81) suggest that the scale maintained excellent internal reliability (45), similar to that reported with the original scale [α = 0.86; (44, 46)]. Respondents answered each item using a 5-point Likert scale ranging from 1 (low) to 5 (high). Two items were reverse-scored, so that the higher the score, the more satisfied the respondent is with their relationship with their dog.

The third part of the survey asked respondents to first rate how often the child in question engaged in 11 behaviors/interactions, which, from here on, we refer to as child-dog interactions. We did not state a specific time period on which to reflect on behaviors. These child-dog interactions were determined based on our previous qualitative research exploring common child-dog interactions that impact on pet dog quality of life (38). Respondents were asked to rate the frequency of each child-dog interaction using a 3-point scale: “never,” “sometimes,” and “often.” The 11 interactions included: angry, cry, tantrums, and meltdowns (henceforth shortened to “meltdowns”), child and dog being in the car together, child visitors at the home, child cuddles and/or kisses the dog, child grooms and/or bathes the dog, child engages in high energy activities with the dog (e.g., running, ball throwing, obstacle courses), child plays with wheeled and/or loud toys (including skates etc.), child disrupts the dogs routine for one reason or another, child is rough with the dog—either accidentally or on purpose (e.g., accidently jumping on them, pulling their tail, cuddling them too tightly) (henceforth referred to as rough contact), child and dog sit quietly together (e.g., reading or watching the TV together), child disturbs their dog—for instance when they are in their bed/safe place (henceforth referred to as disturb safe place). If respondents selected “sometimes” or “often” they were directed to a question which asked them to select which of the previously described 22 behaviors their dog showed during this interaction, including a “none listed” option. As with the well-being measure, a binary 1/0 scoring system was used to promote objectivity.

### Statistical Analysis

Since our research sought to address a number of questions, we used the most appropriate statistical models and software (including SPSS, R Studio, and JASP) for each elements of the analysis. Details of the models used are provided below.

Our initial analysis sought to identify significant differences between those respondents who had a child with a neuro-developmental disorder and those who had a neuro-typically developing child in the raw dependent variables measured. Since our data were non-normally distributed (Shaprio-Wilk, *p* < 0.05), Mann-Whitney U tests were used to examine differences at this level in relation to the dogs' characteristics, total health issues (max score 14), body score (score 1–9, with 4–5 being ideal), the respondents' relationship satisfaction with their dog (based on Relationship Assessment Scale, allowing a max score of 35, higher scores are indicative of greater relationship satisfaction) and the frequency with which the dog and child were reported to engage in each of the interactions. Given the preliminary level of this analysis, the risk of identifying spurious relationships was considered to be of less importance than the risk of failing to identify potentially important relationships for future study; therefore no statistical correction was applied for multiple testing as per the recommendations of Perneger (47).

The second stage of our analysis focused on building composite measures (“behavioral constructs” relating to the dogs' responses) with content validity (i.e., that the construct represents component features); this was guided by the expectation that some of the 22 behaviors assessed would reflect the same underlying latent behavioral construct. Initial explorations of graphed data revealed high correlations between items, suggesting some items should be grouped together. However, due to missing data (resulting from particular situations, such as grooming/bathing the dog, not arising in some families) and multicollinearity between variables, the data did not meet the assumptions for principal components analysis (PCA) nor was it suitable for tetrachoric correlation analysis, or other empirical-based corrections. Therefore, behaviors were grouped into meaningful constructs based solely on Spearman's correlations between items; The internal reliability of these constructs was then assessed using Cronbach Alpha with Chi Square goodness of fit tests. Qualitative theoretical assessment of the content validity of possible constructs was determined by reference to expert group discussions as per Clark and Watson (48).

The third stage of our analysis evaluated the extent to which different child-dog interactions impacted on dog behavior (as assessed by scores on the three constructs identified in the previous stage of analysis) which transcend their developmental group status. In so doing, we potentially identified the most important threats to dog well-being regardless of the nature of the child's developmental pattern. To this end, Friedman tests were computed comparing construct scores separately across the range of child-dog interactions. *Post-hoc* pairwise comparisons were conducted using Wilcoxon *t*-tests to identify where any differences lay.

The next (fourth) stage assessed simply whether dogs showed different construct scores during each type of child-dog interaction depending on whether the interaction involved a neuro-typically developing child or a child with a neuro-developmental disorder (i.e., the two developmental groups), utilizing Mann-Whitney U tests. This analysis was then expanded to consider a wider range of factors in the next stage of analysis.

In the fifth stage we determined whether specific factors predicted owners' reports of their dogs' reactions to specific child-dog interactions using forwards stepwise entry regression models. This allowed us to evaluate what individual factors might be most important to the risk posed by specific interactions. Separate models were computed for each behavioral construct in each child-dog interaction. The factors which were entered as predictor variables included: frequency of interaction (e.g., frequency of meltdowns was entered in to the “meltdowns” model) whether the respondent was the main caregiver for the dog and for the child, child age, the child's developmental status (neuro-developmental disorder or neuro-typically developing), the child primary diagnosis (neuro-developmental disorder group), dog's age, length of time dog owned, training the dog has received, if the dog was a single pure-breed or cross breed, the dog's weight category. Given that our priority was to identify potential predictors of animal well-being, no adjustment to the traditional significance threshold of *p* < 0.05 for the models was made despite the large number built. This is in line with the precautionary principle advised for exploratory research such as this and given that each model may test a separate (47).

The final stage of the analysis assessed whether scores on the quality of life scale developed here showed convergent validity (i.e., associated with other measures of well-being) with other proxies of well-being and relationship satisfaction using regression analysis. Forward stepwise regression analysis was used separately for each construct both across child-dog interactions (i.e., a given construct score across the 11 interactions) and separately for each interaction (i.e., a given construct score for a specific interaction, such as “meltdowns”).

## Results

### Response Rate

Five hundred twenty-three participants started the survey, with 402 fully completed responses (76.9%) taken forward for analysis (neuro-typically developing group, *n* = 194; neuro-developmental disorder group, *n* = 208). Withdrawn responses, defined as those that completed less than half of the survey, tended not to provide initial demographic questions so it is not possible to reliably assess if there were differences in response rates between the two developmental groups (neuro-typical vs. neuro-developmental disorder).

### Sample Characteristics

Sample characteristics are displayed in Table 1. For the large majority of cases the survey respondent was the mother (92.3%) of the child and the primary caregiver of the dog (97.9%). Small to medium sized dogs (54.6%) were slightly more popular than larger dogs (41.2%), with a roughly equal divide in homes that owned a pure, single breed dog (55.2%) compared to a cross, mixed breed dog (44.8%). The most common level/type of training the dogs received was general obedience training (39.2%), or no specific training (39.2%). In homes with neuro-typically developing children the most common primary diagnosis was autism spectrum disorder (74%), followed by attention deficit hyperactivity disorder (ADHD) (13.9%), with all other diagnoses having a prevalence of <2%. Of the final sample (*n* = 402), 293 reported that they found the supporting website “useful” (72.9%), 77 “somewhat useful” (19.2%), 30 “not useful” (7.5%) and 2 declined to answer the question (0.5%). Average completion time for the survey was 21.3 min ± 82 (Mean ± SD).

We also explored for similarities/differences between our two samples (child developmental status: neuro-typically developing and neuro-developmental disorder). There were no statistically significant differences between the two samples on the characteristics reported in Table 1 (all *p's* > 0.05), with the exception of child age. Children in the neuro-typically developing group were significantly younger than children in the neuro-developmental disorder group (*U* = 13,688, *p* < 0.01). No statistically significant differences were observed between the two groups for the dog's overall well-being (total health issues and body score), or relationship with the survey respondent (all *p's* > 0.05). Across the groups there was a trend for the dogs to be perceived as being under rather than over weight. In general respondents reported few symptoms indicative of poor health/ long-term stress and high relationship satisfaction (see Table 1).

The frequency of 11 child-dog interactions are reported in Table 2. Analyses revealed a statistically significant difference, between children with a neuro-developmental disorder and neuro-typically developing children, in the frequency of “angry, crying, meltdowns, and tantrums” (*U* = 12094, *p* < 0.01), and in the frequency of “playing with loud and/or wheeled toys” (*U* = 16615, *p* < 0.01). Neuro-typically developing children had less frequent outbursts of anger, crying, meltdowns, and tantrums than children with a neuro-developmental disorder and were more likely to play with wheeled/noisy toys.

**Table 2:** Frequency of child-dog interactions: percent (%) and total (*n*) data.

	**Neuro-typically developing (*n* = 194)**	**Neuro-developmental disorder (*n* = 208)**
**Angry, cry, meltdown**		
Never	22.7% (44)	3.4% (7)
Sometimes	61.9% (120)	50.5% (105)
Often	15.5% (30)	46.2% (96)
**In car with dog**		
Never	9.3% (18)	10.1% (21)
Sometimes	60.8 (118)	51.9% (108)
Often	29.9% (58)	38% (79)
**Child visitors to home**		
Never	4.6% (9)	10.1% (21)
Sometimes	64.4% (125)	63. (% (133)
Often	30.9% (60)	26% (54)
**Child cuddles/kisses dog**		
Never	8.2% (16)	10.6% (22)
Sometimes	36.1% (70)	26.9% (56)
Often	55.7% (108)	62.5% (130)
**Child grooms/bathes dog**		
Never	56.7% (110)	50% (104)
Sometimes	40.7% (79)	41.3% (86)
Often	2.6% (5)	8.7% (18)
**Child engages in high energy activities with dog**		
Never	6.7% (13)	12% (25)
Sometimes	53.6% (104)	54.3% (113)
Often	39.7% (77)	33.7% (70)
**Child plays with wheeled/noisy toys**		
Never	56.7% (110)	45.2% (94)
Sometimes	37.1% (72)	40.4% (84)
Often	6.2% (12)	14.4% (30)
**Child rough with dog**		
Never	55.2% (107)	51.9% (108)
Sometimes	41.2% (80)	41.8% (87)
Often	3.6% (7)	6.3% (13)
**Child disrupts dog's routine**		
Never	47.4% (92)	43.8% (91)
Sometimes	44.3 (86)	45.2% (94)
Often	8.2% (16)	11.1% (23)
**Child and dog sit quietly together**		
Never	4.1% (8)	8.2% (17)
Sometimes	33.5% (65)	32.7% (68)
Often	62.4% (121)	59.1% (123)
**Child disturbs dog's safe place**		
Never	56.7% (110)	48.1% (100)
Sometimes	37.1% (72)	48.1% (100)
Often	6.2% (12)	3.8% (8)

### Behavioral Constructs

Significant moderate-strong inter-correlations between: repetitive behaviors, bouncing, chewing, vocalizations, ignoring commands, play bow, playing with toy, high tail wag, and responding to commands (*r's* = 0.618–0.993; Table 3) defined the first construct. This construct had excellent internal reliability (α = 0.925, χ^2^ = 46.62, *p* < 0.001). The expert group opinion was that these behaviors best described content validity for assessing the construct of “Excitability.”

**Table 3:** Spearman correlations of behaviors defining the “Excitability” construct.

	**Repetitive**	**Bounce**	**Chew**	**Vocal**	**Ignore**	**Play bow**	**Play toy**	**High tail**	**Respond**
Repetitive	1	0.864**	0.692*	0.817**	0.754**	0.788**	0.644*	0.679*	0.706*
Bouncing		1	0.724y	0.747**	0.737**	0.769**	0.628*	0.697*	0.752**
Chewing			1	0.815**	0.400	0.815**	0.795**	0.732*	0.849**
Vocal				1	0.761**	0.447	0.282	0.373	0.409
Ignore					1	0.690*	0.718*	0.629*	0.637*
Play bow						1	0.963**	0.849**	0.895**
Play toy							1	0.843**	0.879**
High tail								1	0.964*
Respond									1

* p < 0.05;

** *p < 0.01*.

Significant moderate-strong inter-correlations between: moving close to child, laying down/relaxed, and yawning (*r's* = 0.738–0.805; Table 4) created the second construct. This construct had good internal reliability (α = 0.753, χ^2^ = 12.45, *p* < 0.01). The expert group discussions concluded that these behaviors show best content validity for assessing the construct of “Calmness.”

**Table 4:** Spearman correlations of behaviors defining the “Calmness” construct.

	**Close to child**	**Laying down/relaxed**	**Yawning**
Close to child	1	0.800**	0.690*
Laying down/relaxed		1	0.712*
Yawning			1

* p < 0.05;

** *p < 0.01*.

In the opinion of the expert group, the remaining items appeared to be indicative of “Fearfulness” and included: panting, listless, cowering, lip/nose lick, wide eyes, shaking, running away, child-directed aggression, hiding, seeking safety. Correlations between these items were more varied (*r's* = –0.041 to 0.925; Table 5), but each element showed at least one significant relatively strong correlation with another. The negative correlations appeared to relate to either alternative behavior strategies based on context (e.g., running away vs. child aggression) or differences in intensity based on context (e.g., running away vs. panting). Our judgement to keep the items together as a single construct was supported by its excellent internal reliability (α = 0.863, χ^2^ = 33.91, *p* < 0.001).

**Table 5:** Spearman correlations of behaviors defining the “Fearfulness” construct.

	**Panting**	**Listless**	**Cowering**	**Lip/nose lick**	**Wide eyes**	**Shaking**	**Run away**	**Child aggression**	**Hiding**	**Seek safety**
Panting	1	0.201	0.455	0.689*	0.368	0.626*	−0.041	0.126	0.087	0.404
Listless		1	0.701*	0.310	0.689	0.451	0.539	0.132	0.689*	0.691*
Cowering			1	0.717*	0.908**	0.734*	0.761**	0.301	0.593	0.701*
Lip/nose lick				1	0.819*	0.413	0.342	0.469	0.280	0.523
Wide eyes					1	0.471	0.776**	0.576	0.615*	0.606*
Shaking						1	0.444	−0.037	0.423	0.507
Run away							1	0.526	0.816**	0.812**
Child aggression								1	0.516	0.352
Hiding									1	0.795**
Seek safety										1

* p < 0.05;

** *p < 0.01*.

Henceforth, for clarity, the capitalized name-labels identified above is used to refer to these constructs. The instrument which assesses these in order to evaluate the dog's quality of life is referred to as the Lincoln Pet dog Quality of Life scale (Lincoln P-QOL). The Lincoln P-QOL is devised of items which assess the behavioral constructs of “Excitability,” “Fearfulness,” and “Calmness.”

### The Effect of Child-Dog Interactions on Pet Dog Quality of Life Scores

As expected, specific child-dog interactions had varying (positive and negative) effects on the constructs within the Lincoln P-QOL scores.

#### Excitability

There was a significant difference between scores on Excitability across the child-dog interactions χ^2^ (10) = 125.9, *p* < 0.001 (Table 6, see also Supplementary Table 1). Further comparisons revealed a degree of hierarchy to the scores based on activity. Scores were significantly higher during child-dog high energy activities compared to all other interactions (*p's* < 0.01), and significantly higher when child visitors were in the home to all other interactions with the exception of these high energy activities (all other *p's* < 0.01). Excitability scores were also higher when the child played with loud/wheeled toys compared to all other interactions with the exception of high energy activities, cuddling/kissing and high energy activities (all other *p's* < 0.01). Excitability scores were significantly higher for cuddling and kissing compared to all other interactions (*p's* < 0.01) excluding child visitors, high energy, and loud/wheeled toys. Excitability scores were significantly lower when the child and dog were in the car together compared to all other interactions (*p's* < 0.01).

**Table 6:** Descriptive statistics illustrating the impact of various types of child-dog interaction on each element of the Lincoln Pet Dog Quality of Life scale scores.

**Groups combined**	**Quality of life indices**								
**Child-dog interaction**	**Excitability**			**Calmness**			**Fearful**		
	**Mean ± SE^a^**	**IQR^b^**	**Median**	**Mean ± SE**	**IQR**	**Median**	**Mean ± SE**	**IQR**	**Median**
Angry, cry, meltdown	0.84 ± 0.19	2.00	0.50	1.15 ± 0.12	1.00	1.00	0.81 ± 0.23	1.00	0.00
In car together	0.01 ± 0.01	0.00	0.00	0.46 ± 0.16	1.00	1.00	0.65 ± 0.22	1.00	0.00
Child visitors to home	3.07 ± 0.28	2.25	3.00	1.03 ± 0.14	1.25	1.00	0.27 ± 0.14	0.00	0.00
Cuddles/kisses	1.61 ± 0.33	2.25	1.00	1.76 ± 0.12	1.00	2.00	0.57 ± 0.24	1.00	0.00
Grooms/bathes	0.92 ± 0.17	1.00	1.00	0.76 ± 0.15	1.00	1.00	0.88 ± 0.25	1.00	0.00
High energy activities	3.92 ± 0.36	2.00	4.00	0.69± 0.15	1.00	1.00	0.15 ± 0.07	0.00	0.00
Wheeled/noisy toys	1.57 ± 0.40	3.25	1.00	0.69 ± 0.12	1.00	1.00	0.28 ± 0.11	0.00	0.00
Child rough with dog	0.76 ± 0.21	1.25	0.00	0.46 ± 0.11	1.00	0.00	1.04 ± 0.31	2.00	0.00
Disrupts dog's routine	1.11 ± 0.30	1.25	1.00	0.57 ± 0.12	1.00	0.50	0.55 ± 0.24	1.00	0.00
Sit quietly together	0.57 ± 0.13	1.00	0.00	1.92 ± 0.12	0.00	2.00	0.26 ± 0.13	0.00	0.00
Disturbs safe place	0.91 ± 0.21	2.00	0.50	0.84 ± 0.15	1.25	1.00	0.73 ± 0.29	1.00	0.00
**Typically developing**	**Quality of life indices**								
**Child-dog interaction**	**Excitability**			**Calmness**			**Fearful**		
	**Mean** **±** **SE**	**IQR**	**Median**	**Mean** **±** **SE**	**IQR**	**Median**	**Mean** **±** **SE**	**IQR**	**Median**
Angry, cry, meltdown	0.45 ± 0.20	1.00	0.00	1.09 ± 0.16	0.00	1.00	0.54 ± 0.20	1.00	0.00
In car together	0.01 ± 0.01	0.00	0.00	0.72 ± 0.23	1.00	1.00	0.45 ± 0.15	1.00	0.00
Child visitors to home	2.54 ± 0.49	3.00	2.00	1.09 ± 0.25	2.00	1.00	0.18 ± 0.12	0.00	0.00
Cuddles/kisses	0.91 ± 0.36	1.00	1.00	1.63 ± 0.24	1.00	2.00	0.63 ± 0.24	1.00	0.00
Grooms/bathes	0.36 ± 0.15	1.00	0.00	1.09 ± 0.28	2.00	1.00	0.72 ± 0.35	1.00	0.00
High energy activities	3.72 ± 0.52	2.00	4.00	0.54 ± 0.20	1.00	0.00	0.09 ± 0.09	0.00	0.00
Wheeled/noisy toys	1.54 ± 0.56	4.00	1.00	0.63 ± 0.15	1.00	1.00	0.09 ± 0.09	0.00	0.00
Child rough with dog	0.63 ± 0.31	1.00	0.00	0.45 ± 0.15	1.00	0.00	0.82 ± 0.29	1.00	1.00
Disrupts dog's routine	1.00 ± 1.27	1.00	0.00	0.72 ± 0.19	1.00	1.00	0.09 ± 0.09	0.00	0.00
Sit quietly together	0.36 ± 0.15	1.00	0.00	1.81 ± 0.22	0.00	2.00	0.36 ± 0.27	0.00	0.00
Disturbs safe place	0.72 ± 0.33	2.00	0.00	1.00 ± 0.23	2.00	1.00	0.72 ± 0.38	1.00	0.00
**Neuro-developmental disorder**	**Quality of life indices**								
**Child-dog interaction**	**Excitability**			**Calmness**			**Fearful**		
	**Mean** **±** **SE**	**IQR**	**Median**	**Mean** **±** **SE**	**IQR**	**Median**	**Mean** **±** **SE**	**IQR**	**Median**
Angry, cry, meltdown	1.13 ± 0.29	2.00	1.00	1.20 ± 0.17	1.00	1.00	1.00 ± 0.37	2.00	0.00
In car together	0.01 ± 0.01	0.00	0.00	0.86 ± 0.23	1.00	1.00	0.80 ± 0.36	2.00	0.00
Child visitors to home	3.46 ± 0.31	3.00	3.00	1.00 ± 0.16	0.00	1.00	0.33 ± 0.23	0.00	0.00
Cuddles/kisses	2.21 ± 0.48	3.00	2.00	1.86 ± 0.13	0.00	2.00	0.53 ± 0.40	0.00	0.00
Grooms/bathes	1.33 ± 0.23	1.00	0.00	0.53 ± 0.16	1.00	0.00	1.00 ± 0.36	2.00	0.00
High energy activities	4.06 ± 0.52	2.00	4.00	0.80 ± 0.22	1.00	1.00	0.20 ± 0.11	0.00	0.00
Wheeled/noisy toys	1.60 ± 0.58	3.00	0.00	0.73 ± 0.18	1.00	1.00	0.40 ± 0.19	1.00	0.00
Child rough with dog	0.86 ± 0.31	2.00	0.00	0.46 ± 0.16	1.00	0.00	1.13 ± 0.49	2.00	0.00
Disrupts dog's routine	1.20 ± 0.39	2.00	1.00	0.47 ± 0.16	1.00	0.00	0.80 ± 0.40	1.00	0.00
Sit quietly together	0.73 ± 0.21	1.00	1.00	2.00 ± 0.13	0.00	2.00	0.70 ± 0.11	0.00	0.00
Disturbs safe place	1.13 ± 0.29	2.00	1.00	0.73 ± 0.21	1.00	1.00	0.73 ± 0.44	0.00	0.00

See text for details of significant differences.

a Standard Error.

b *Inter-Quartile Range*.

#### Calmness

There was a significant difference between scores on Calmness across the child-dog interactions χ^2^ (10) = 94.8, *p* < 0.001 (Table 6, see also Supplementary Table 1), with a hierarchy emerging between four of the activities, which scored significantly higher than the rest. Scores on Calmness were significantly higher during the child and dog spending quiet time together compared to all other interactions (*p's* < 0.01), and were significantly higher when the child cuddled/kissed the dog compared to all other interactions (*p's* < 0.01), with the exception of “quiet time.” Scores on Calmness were also comparatively high during meltdowns and having child visitors to the home, being significantly higher than in all other interactions (*p's* < 0.05), with the exception of the two aforementioned—and being higher in relation to meltdowns than child visitors (*p* < 0.01).

#### Fearfulness

There was a significant difference between scores on Fearfulness across the child-dog interactions χ^2^ (10) = 32.3, *p* < 0.001 (Table 6, see also Supplementary Table 1). Scores on Fearfulness were significantly higher during rough contact between the child and dog compared to all other child-dog interactions (*p's* < 0.01). Scores were second highest during meltdowns, being significantly higher than that observed with most child-dog interactions, with the exception of rough contact and grooming/bathing. Fearfulness scores were third highest on grooming/bathing, being significantly higher than other child-dog interactions with the exception of rough contact, meltdowns, and playing with loud/wheeled toys. Scores on Fearfulness were lowest on child and dog playing high energy games together and spending quiet time together.

### Comparison of Dog Responses From Families With Neuro-Typically Developing Children and Children With Neuro-Developmental Disorders

Respondents reported few differences in the scores of dogs on the behavioral constructs for the various child-dog interactions based simply on whether the interaction in question involved a neuro-typically developing child or a child with a neuro-developmental disorder (Table 6). Dogs living with children with a neuro-developmental disorder scored significantly higher on Excitability during both meltdowns (*U* = 13560, *p* < 0.05) and bathing/grooming (*U* = 3713, *p* < 0.05) and on Fearfulness during “quiet time spent with the dog and child” (*U* = 16120, *p* < 0.01). No other comparisons were statistically significant at *p* < 0.05.

### Predictors of Pet Dog Quality of Life in Different Activities

In general, the demographic data used in the multivariate models explained only a small amount of the variance in any of the constructs in the situations examined (maximum adjusted R-squared value was 0.08).

#### Child Is Accidently or on Purpose, Rough With the Dog

Predictors could be found only for the construct of Excitability within the Lincoln-PQOL, i.e., there were no statistically significant predictors of Calmness and Fearfulness during this situation. At 8%, the final model accounted for the highest proportion of the variance within any construct for any activity (*F* = 6.27, *p* < 0.01, Adj *R*^2^ = 0.08). Three factors were included in the model building process in the following order: whether the dog was a single breed or not (β = 0.23, *t* = 3.22, *p* < 0.01, *F* = 8.73, Adj *R*^2^ = 0.04), with higher scores associated with single (pure) breed dogs; child age (β = 0.15, *t* = 2.10, *p* < 0.04, *F* = 7.33, increasing Adj *R*^2^ = 0.06), with higher scores associated with older children; whether the respondent was the main caregiver for the dog or not (β = −0.14, *t* = −1.99, *p* < 0.05, *F* = 6.28, increasing final Adj *R*^2^ = 0.08), if the respondent was the main caregiver for the dog they perceived less Excitability behaviors in the dog.

#### Child Disturbs the Dog (e.g., When in Safe Place)

Predictors were identified for both Excitability and Calmness of the dog in relation to this situation, but there were no statistically significant predictors of Fearfulness. Seven percentage of the variance in Excitability score when the child disturbed the dog was explained by the single factor of whether or not the respondent was the main caregiver for the child or not (β = −0.27, *F* = 15.30, *p* < 0.001, Adj *R*^2^ = 0.07), with lower scores associated with the respondent being the child's main caregiver. Six percentage of the variance in Calmness during this situation was associated with the weight category of the dog (β = 0.25, *F* = 13.01, *p* < 0.001, Adj *R*^2^ = 0.06), with larger weight dogs associated with higher Calmness scores when this occurred.

#### Meltdowns

Predictors were identified only in relation to the construct of Excitability by the dog. The final model accounted for 7% of the variance (*F* = 9.93, *p* < 0.001, Adj *R*^2^ = 0.07) and included three variables: length of time the dog was owned (β = −0.19, *t* = −3.76, *p* < 0.001, *F* = 13.18, Adj *R*^2^ = 0.03), respondent was the main caregiver for child (β = −0.16, *t* = −3.07, *p* < 0.003, *F* = 12.51, increasing Adj *R*^2^ to 0.06) and main caregiver for dog (β = −0.11, *t* = −2.13, *p* < 0.05, *F* = 9.93, finally increasing Adj *R*^2^ to 0.07). Thus, the longer the dog had been owned the lower the level of Excitability reported during meltdowns. If the respondent was the main caregiver of the child or to the dog they perceived the dog as showing less Excitability behaviors during meltdowns. The significant relationship with neurodevelopmental status of the child, identified in the simple test of association undertaken in the previous section was lost in this multivariate analysis.

#### Child Visitors to the Home

Significant predictors were identified for both Excitability and Fearfulness scores but not Calmness. The model for Excitability included two variables and accounted for 6% of the variance (*F* = 12.00, *p* < 0.001, Adj *R*^2^ = 0.06): the longer the dog had been owned the lower the Excitability score when children visited the home (β = −0.23, *t* = −4.43, *p* < 0.001, *F* = 19.78, Adj *R*^2^ = 0.05); the older the child the higher the Excitability score (β = 0.10, *t* = 2.01, *p* < 0.05, *F* = 12.00, increasing Adj *R*^2^ to 0.06). Fearfulness behaviors when child visitors were at the home (*F* = 5.27, *p* < 0.007, Adj *R*^2^ = 0.02) were related to dog weight category (β = −0.14, *t* = −2.63, *p* < 0.01, *F* = 6.08), and the respondent being main caregiver to the dog (β = −0.11, *t* = −2.10, *p* < 0.05, *F* = 5.27), each contributing 1% to the variance explained. Lower weight dogs tended to respond with higher Fearfulness, while the respondent being the main caregiver to the dog was associated with reporting of less Fearfulness.

#### Child Cuddles/Kisses the Dog

Models could be built for all three constructs in relation to this activity. Excitability (*F* = 4.91, *p* < 0.01, Adj *R*^2^ = 0.02) was predicted by child age (β = 0.13, *t* = 2.43, *p* < 0.02, *F* = 5.81), and dog age (β = −0.10, *t* = −1.99, *p* < 0.05, *F* = 4.91), each contributing 1% to the variance explained. Dogs were reported to show more Excitability when older children cuddle/kiss them and when the dogs were younger. Calmness was predicted by the length of ownership of the dog (β = −0.12, *F* = 4.92, *p* < 0.03, Adj *R*^2^ = 0.01), with calmer dog behavior associated with a shorter time of ownership. Fearfulness was associated with three variables (*F* = 7.01, *p* < 0.01, Adj *R*^2^ = 0.06): child age (β = −0.17, *t* = −3.23, *p* < 0.001, *F* = 9.29, Adj *R*^2^ = 0.02), dog weight (β = −0.15, *t* = −2.85, *p* < 0.01, *F* = 8.43, increasing Adj *R*^2^ to 0.04), and dog age (β = 0.10, *t* = 2.01, *p* < 0.05, *F* = 7.01, further increasing Adj *R*^2^ to 0.06). The relationships indicate that Fearfulness behaviors during cuddling and kissing are higher when the child is younger, when the dog is of a lower body weight and when the dog is older.

#### Child and Dog Spend Quiet Time Together

Models could be built for all three constructs in relation to this activity. Excitability when the child and dog spend quiet time together was related only to child age (β = 0.18, *F* = 11.84, *p* < 0.001, Adj *R*^2^ = 0.03), with higher scores associated with older children. Calmness was related only to dog weight category (β = −0.15, *F* = 8.32, *p* < 0.01, Adj *R*^2^ = 0.02), with smaller dogs showing higher Calmness. Fearfulness was related to child's developmental status and whether or not the respondent was the main caregiver for the dog or not (*F* = 5.38, *p* < 0.01, Adj *R*^2^ = 0.02). Child's developmental status (β = 0.12, *t* = 2.34, *p* < 0.03, *F* = 6.15), and whether or not the respondent was the main caregiver for the dog (β = −0.11, *t* = −2.13, *p* < 0.04, *F* = 6.15 each explained 1% of the variance. Dogs appeared to demonstrate higher Fearfulness behaviors when they spent quiet time with a child with a neuro-developmental disorder and if the respondent was not the main caregiver to the dog.

#### Child Plays With Loud/Wheeled Toys

Excitability scores when the child played with loud/wheeled toys were related to pedigree status (β = 0.14, *F* = 5.29, *p* < 0.03, Adj *R*^2^ = 0.02), with dogs who were single/pure breed appearing to express higher Excitability when the child played with loud/wheeled toys. Fearfulness was associated with dog age (β = 0.19, *F* = 9.05, *p* < 0.01, Adj *R*^2^ = 0.03), with older dogs having higher scores. No significant models were identified for Calmness.

#### Child Grooms/Bathes the Dog

Only Excitability scores when the child groomed/bathed the dog were related to any of the demographic variables considered (β = −0.17, *F* = 5.71, *p* < 0.02, Adj *R*^2^ = 0.03), and only one variable was useful in this regard. As in the simpler univariate analysis, dogs appeared to show higher Excitability when children with neuro-developmental disorders groomed/bathed them.

#### Child Engages in High Energy Activities With the Dog

Excitability scores were related only to child age (β = 0.15, *F* = 8.25, *p* < 0.01, Adj *R*^2^ = 0.02), with higher Excitability related to high energy activities with older children. Fearfulness scores were only related to whether the respondent was the main caregiver to the child or not (β = −0.12, *F* = 5.45, *p* < 0.01, Adj *R*^2^ = 0.01), with main caregivers for the child perceiving less Fearfulness during high energy activities. No significant models were identified for Calmness.

#### Child Disrupts the Dog's Routine

Excitability when the child disrupts the dog's routine was only related to the child's age (β = 0.15, *F* = 5.01, *p* < 0.03, Adj *R*^2^ = 0.02), with higher Excitability associated with disruption by older children. Calmness, was however related only to the dog's age (β = −0.14, *F* = 4.20, *p* < 0.03, Adj *R*^2^ = 0.01), with younger dogs showed higher Calmness. No significant models were identified for Fearfulness.

#### Child and Dog in the Car

There were no statistically significant predictors for Excitability, Calmness and Fearfulness when the child and dog were in the car together.

### Convergent Validity of the Constructs

The Lincoln P-QOL showed convergent validity with several of the other proxies of well-being including relationship satisfaction as outlined below.

#### Stress Related Health Issues

##### Construct totals

Only the total score for Fearfulness predicted the individual variability observed in the dog's total score for the stress related health issues (β = 0.36, *F* = 57.59, *p* < 0.001, Adj *R*^2^ = 0.12) across all interactions. Higher Fearfulness scores predicted a higher number of health issues. Excitability and Calmness scores were not predictive at this level.

##### Specific interactions

A significant predictive relationship between Fearfulness and health scores was also evident in ten of the eleven models of specific child-dog interactions; the relationship was only insignificant for when the child played with loud or wheeled toys (*Child is rough with the dog:* β = 0.37, *F* = 28.78, *p* < 0.01, Adj *R*^2^ = 0.13; *Child visitors in the home:* β = 0.33, *F* = 44.81, *p* < 0.001, Adj *R*^2^ = 0.11; *Child groomed/bathed the dog:* β = 0.30, *F* = 18.49, *p* < 0.001, Adj R^2^ = 0.09; *Child disrupts the dog's routine:* β = 0.25, *F* = 14.10, *p* < 0.01, Adj *R*^2^ = 0.06; *Child and dog in the car:* β = 0.23, *F* = 20.11, *p* < 0.001, Adj *R*^2^ = 0.05; *Child cuddled/kissed the dog:* β = 0.22, *F* = 17.98, *p* < 0.001, Adj *R*^2^ = 0.05; *Child and dog spend quiet time together* β = 0.22, *F* = 18.61, *p* < 0.01, Adj *R*^2^ = 0.05; *Child disturbs the dog's safe place:* β = 0.19, *F* = 7.23, *p* < 0.01, Adj *R*^2^ = 0.03; *Child engages the dog in high energy activities:* β = 0.15, *F* = 7.97, *p* < 0.01, Adj *R*^2^ = 0.02). In the case of *meltdowns*, Excitability scores also featured in the model predicting stress related health scores (*F* = 11.80, *p* < 0.001, Adj *R*^2^ = 0.06, with fearfulness predicting 5% of the variance and Excitability a further 1%). Calmness scores did not predict total health issues in any model.

#### Body Condition Score

##### Construct totals

Higher Excitability total score was associated with a lower body condition score (β = −0.15, *F* = 8.65, *p* < 0.01, Adj *R*^2^ = 0.02). The relationship with the other two constructs (Feafulness and Calmness) was not significant.

##### Specific interactions

The relationship between Excitability and dog body condition score was reflected in models of two specific child-dog interactions: *Child grooms/bathes the dog* (β = −0.16, *F* = 5.09, *p* < 0.03, Adj *R*^2^ = 0.02) and *Child disrupts the dog's routine*. (β = −0.18, *F* = 7.52, *p* < 0.01, Adj *R*^2^ = 0.03).

#### Relationship Satisfaction

##### Construct totals

Calmness total score and Fearfulness total score but not Excitability total score significantly contributed to owner relationship satisfaction with their dog (*F* = 18.95, *p* < 0.001, Adj *R*^2^ = 0.08). Each accounted for about 4% of the variance in the final model (Calmness total score β = 0.21, *t* = 4.47, *p* < 0.001; Fearfulness total score β = −0.21, *t* = −4.36, *p* < 0.001). As Calmness scores increased and Fearfulness scores decreased, relationship satisfaction increased.

##### Specific interactions

The positive relationship between Calmness scores and relationship satisfaction was also apparent in models relating to four specific child-dog interactions, namely: the child being rough with the dog, meltdowns, the child grooming/bathing the dog and the child disrupting the dog's routine. The model for meltdowns also included a negative relationship between Excitability scores and owner-relationship satisfaction. Excitability scores did not feature in any other models. The negative relationship between Fearfulness scores and relationship satisfaction was apparent in models relating to five specific child-dog interactions, namely: the child cuddling/kissing the dog, the child and dog spending quiet time together, the child grooming/bathing the dog, the child disrupting the dog's routine as well as the child and dog being in the car together. Details of the model fitting process are described below.

*Child being rough with the dog*. Scores on Calmness when the child was rough with the dog was a significant predictor of variance in relationship satisfaction, accounting for 2% of individual variability (β = 0.16, *F* = 4.62, *p* < 0.04, Adj *R*^2^ = 0.02).

*Meltdowns*. A model based on two variables, Calmness and Excitability during meltdowns accounted for 4% variance of the variability observed in owner relationship satisfaction with their dog in relation to child meltdowns (*F* = 7.59, *p* < 0.001, Adj *R*^2^ = 0.04). Calmness entered first into the model (β = 0.21, *t* = 3.69, *p* < 0.001, *F* = 8.63, Adj *R*^2^ = 0.02), and Excitability entered second (β = −0.14, *t* = −2.53, *p* < 0.01, *F* = 7.59, Adj *R*^2^ = 0.04).

*Child cuddling/kissing the dog*. Scores on Fearfulness when the child cuddled/kissed the dog was a significant predictor of variance in relationship satisfaction, accounting for 2% of individual variability (β = −0.15, *F* = 7.82, *p* < 0.01, Adj *R*^2^ = 0.02).

*Child and dog spending quiet time together*. Scores on Fearfulness when the child and dog were spending quiet time together was a significant predictor of variance in relationship satisfaction, accounting for 2% of individual variability (β = −0.13, *F* = 6.67, *p* < 0.02, Adj *R*^2^ = 0.02).

*Child grooming/bathing the dog*. Fearfulness scores during the child grooming/bathing the dog and Calmness scores during this interaction were included in the final model, accounting for 7% of the variance in the owner's relationship satisfaction with their dog (*F* = 7.45, *p* < 0.001, Adj *R*^2^ = 0.07). Fearfulness entered first into the model (β = −0.18, *t* = −2.48, *p* < 0.01, *F* = 8.57, Adj *R*^2^ = 0.04), and Calmness entered second (β = 0.18, *t* = 2.47, *p* < 0.01, *F* = 7.45, Adj *R*^2^ = 0.07).

*Child disrupting the dog's routine*. Fearfulness scores and Calmness scores when the child disrupts the dog's routine significantly predicted and accounted for 5% of the variance in owner's relationship satisfaction with their dog (*F* = 6.17, *p* < 0.01, Adj *R*^2^ = 0.05). Calmness entered first into the model (β = 0.18, *t* = 2.78, *p* < 0.01, *F* = 7.94, Adj *R*^2^ = 0.03), and Fearfulness entered second (β = −0.14, *t* = −2.07, *p* < 0.05, *F* = 6.17, Adj *R*^2^ = 0.05).

*Child and dog in the car*. Scores on Fearfulness when the child and dog were in the car together was a significant predictor of variance in relationship satisfaction, accounting for 6% of individual variability (β = −0.24, *F* = 7.59, *p* < 0.001, Adj *R*^2^ = 0.04).

## Discussion

Given increasing interest in the value of pets, particularly dogs, to child well-being, we were motivated to explore the child-dog relationship from the dog's perspective. The Lincoln P-QOL is the first validated scale that can be used to evaluate pet dog quality of life in child-dog interactions. The scale is comprised of three constructs, representing possible positive (Calmness) and negative states (Excitability and Fearfulness). We highlight how some interactions such as considerate child-dog physical affection and spending quiet time together may be beneficial to dog quality of life, whereas other situations, like rough contact and child meltdown may have a negative impact. In general, there appears to be little difference in dog quality of life between those living with a child who is neuro-typically developing and those living with a child with a neuro-developmental disorder. Although the scale has been developed in the context of families, but may also be of value within a wider range of settings, including animal assisted interventions.

### Validity of the Lincoln P-Qol

It is important to note that the definition of the behavioral constructs was derived from expert opinion of the identified behavioral correlates and thus have only face validity. They have not been subject to any other assessment of their content validity at either the level of their valence, intensity of quality, although correlates with other measures taken as part of this study should be noted and are discussed further below. Nonetheless, we emphasize the need for caution in assuming any relationship between the terms as used here for the constructs (which are, in effect, a shorthand for the behavioral correlates revealed here) and other uses of these terms in both the popular and scientific literature. In particular, we the emotional valence of the constructs relating to excitability and calmness, should not be assumed.

The scale grouped a range of dog behaviors into three constructs considered to represent Excitability, Calmness, and Fearfulness. Although data were not suitable for conventional component/factor analysis, correlations between the items still guided the development of these; the reliability and validity of which were confirmed by the statistical evaluation of internal reliability (construct validity), expert group discussions (content validity), and statistical relationships with other proxies of well-being (convergent validity) provided by respondents. Even though the proportion of variance explained by the latter might be considered low, they were significant and the relationship with the small number of demographic variables assessed here was comparable to, and at their best exceeded, that described for predicting other complex responses like human directed aggression [e.g., (49), who used a much larger number of potential predictors]. The analyses support the proposition that Excitability and Fearfulness reflect behavioral responses which are associated with a negative impact on dog well-being and the owner-dog relationship. The Calmness construct, although generally relating to more positive behavior responses which have a lower impact on dog well-being, is potentially more complex. Calmness included yawning, which has previously been described as a stress related behavior or “calming signal” (50). The association of this response with the dog choosing to be close to the child and lying down relaxed, might indicate that it is indeed a signal used to encourage de-arousal, and so may not necessarily indicate poor well-being as has been implied by others [e.g., (27)]. This suggestion is further supported by the finding that there were no health related correlates with this construct. However, we point out that this research, and the statistical approaches taken, were exploratory; this should be taken into consideration when interpreting the results and be used to guide further research in this area.

For the purpose of the initial development of the scale proposed here we did not ask owners to record the presence/absence of behavior over a pre-defined time period, instead we relied on their general observations. This alternative approach should be considered in future work to validate this scale. Indeed, owner-completed scales can be subject to perceptual biases, but this is not necessarily the case and its impact needs to be carefully considered. Nonetheless, it should be recognized that many assessments of animal well-being rely on subjective assessments (19). Interestingly, whether or not the respondent was the main caregiver of either the child or dog had a significant effect on the perceived Excitability and Fearfulness of the dog in 6 of the 11 scenarios, perhaps suggesting that familiarity with the situation may be an important factor in the evaluation of dogs' behavior responses. This potentially has wider implications for those responsible for monitoring dog behavior in stressful situations and deserves further scientific considerations. Indeed, it has previously been noted that even owners may frequently lack knowledge, or awareness, of subtle behaviors and emotional states, and often refer to holistic states (51–54). To help mitigate the impact of this in the current study, we provided a web-based resource, which owners were required to confirm they had viewed, before taking part in the study. Additionally, instead of asking owners to recognize emotional states (e.g., calmness *per se*), or to rate the intensity of an emotion/behavior, we used a binary system whereby owners indicated whether or not they had observed a particular behavior during specific child-dog interactions (42). The emotional constructs then emerged from the subsequent analysis of the relationship between behaviors and expert evaluation of this. Although using binary scoring system has advantages in terms of objectivity, we recognize that recording information surrounding frequency and intensity may lead to a more detailed insight into dog quality of life, this a consideration for future research. Important next steps also include both inter- and intra-rater reliability of the instrument; as well as trained and untrained observers and members in the same household, to identify specific limitations to its application. Additionally, although a web-based resource was developed to help parents recognize their dog's behavior, we did not provide further explanation as to what parents categorized their child's behavior as, for example, caregivers of neuro-typically developing children may classify a meltdown very differently from caregivers of children with a neuro-developmental disorder. Future research should consider defining these behaviors for research purposes.

A notable finding from this work, is how few of the construct scores were consistently related to the neurodevelopmental status of the child. Only bathing/ grooming the dog (which appears to result in a more excitable response) and quiet times (which tend to result in higher fearfulness) tended to be consistently worse when the child had a neurodevelopmental disorder. These specific findings could reflect children with neuro-developmental disorders often having difficulties in organizing action toward a goal, with poor motor co-ordination (55, 56). Additionally, children with neuro-developmental disorders, notably autism spectrum disorder, often have problems with emotion regulation, demonstrating greater emotional reactivity than neuro-typically developing children (57, 58), which may heighten the unpredictability of the course of events when the child is close. Therefore, it seems reasonable to suggest that careful supervision is particularly important at these times. All children may pose a risk to the dog, if they do not interact with it in an appropriate way. Indeed, in relation to Excitability, child age was a predictor in six types of interaction, with older children creating more excitability in the dog. Further research is necessary to determine how a child's interaction style with a dog changes with time to produce this effect, factors might include the duration of interaction and/or its quality.

### Excitability

Scores on this construct were highest in child-dog interactions which may typically involve high levels of auditory (e.g., child noise levels), visual (e.g., novel objects/people), or tactile (e.g., close physical child-dog contact) stimulation; being highest for high energy activities, child visitors, loud/wheeled toys, and cuddling/kissing the dog. This is consistent with sensory stimulation increasing arousal levels in the dog, as they process and react to the stimuli. However, with the exception of excitability during meltdowns, these did not appear to affect either the relationship between the respondent and the dog or the risk to the dog of health-related problems. This co-relationship with meltdowns may reflect several different scenarios, which require further investigation. For example, it might be that a more excitable dog is perceived as a problem and difficult to manage, and the wider stress associated with a breakdown in the relationship results in increased risk of health-related problems (exacerbated by fearfulness in the dog). Alternatively, it might be that meltdowns are particularly stressful for fearful dogs who become excitable as a result and this prolonged stress results in these health-related problems (26), which alone, or together with the dog's reactivity, impacts the relationship. Either way, these results indicate that meltdowns by children (regardless of their neurodevelopmental status, given that this dropped out of the multivariate analysis) may pose one of the biggest potential threats to dog quality of life in a home with children.

A shorter length of time the dog had lived with the child was associated with higher Excitability in meltdowns and with child visitors, with dog age similarly related to Excitability scores during cuddling/kissing. These results might indicate that over time dogs may become accustomed to these interactions, rather than sensitized. However, this could also be interpreted to highlight the importance of paying especially close attention during the initial times when these events occur. It should be noted that the frequency of events, was not predictive of any of the construct scores, so the effect would not seem to be related just to the number of exposures. Attention to the dog's reaction during meltdowns may be particularly important as excitability scores was the only interaction in which, importantly, Excitability predicted owner-relationship satisfaction; higher Excitability scores were associated with lower relationship satisfaction, perhaps because meltdowns are often particularly stressful for parents to manage and the dog displaying Excitability related behaviors during this time will increase parent stress. Research has previously reported that dog excitability is negatively associated with owner attachment (71) and that behaviors which may be demonstrative of excitability (running outdoors, destructive behaviors) affect dog-owner relationship satisfaction (59).

### Calmness

As expected, scores for Calmness were highest during what would be predicted to be gentle child-dog interactions, such as spending quiet time together and cuddling/kissing. However, Calmness scores in child meltdowns were also surprisingly high. This might be explained by several factors: first dogs who do not remain calm, might be rehomed, secondly the composite behaviors of this construct might not reflect that the dog is calm, but that the dog is trying to encourage derousal. For instance, yawning may not only reflect relaxation, but it may also be an active attempt to maintain low arousal (60, 61). Interestingly, the dog moving close to the child during meltdowns is something that has been reported previously to potentially reflect attention-seeking and arousal (38). Therefore, caution is warranted in using individual behaviors to infer the emotional state of a dog (62), and the use of a scale such as that described here, may be more robust.

Human centered demographics did not appear to be important in predicting Calmness, but dog centered demographics were. Owning the dog for less time was associated with higher Calmness scores during cuddling/kissing, perhaps these interactions are done more cautiously, when the two are less familiar with each other, creating a more relaxing experience for the dog. Younger dogs were reported to be calmer during disruption to routine, the reasons for this are unclear, but they could reflect a greater tolerance in younger dogs as routines are less well-established, or a more general reduction in cognitive flexibility associated with aging as has recently been reported in chimpanzees (63). Dogs who weighed less showed higher Calmness during quiet time with the child and lower Calmness when they were disturbed by the child. These results may indicate that quiet time together and time alone without being disturbed may be particularly important for small dogs, if they are to remain calm.

Although Calmness scores were important predictors of owner relationship satisfaction, they were not reliable predictors of well-being such as health issues or body score. In general, relationship satisfaction had a positive relationship with Calmness scores, and this relationship was also observed in a wide range of specific interactions including, grooming/bathing, disrupt routine, rough contact, and meltdowns. It is worth noting that during meltdowns high scores on Calmness and low scores on Excitability predict higher owner relationship satisfaction, indicating this sort of response may be particularly important to owners. This is further evidenced in the re-homing literatures, which indicates that problematic behaviors at critical times are strongly associated with dog relinquishment (25).

### Fearfulness

As predicted, scores on Fearfulness were significantly higher during child-dog interactions which pose a potential physical threat to the dog, including rough contact, meltdowns and grooming/bathing. Scores were lowest on child and dog playing high energy games together and spending quiet time together. These add to the validity of the construct. Scores on the Fearfulness during the child and dog spending quiet time together was significantly higher if the child had a neuro-developmental disorder This might be because children with neuro-developmental disorders, such as autism and ADHD often have difficulties with executive functions such as vigilance and response inhibition (64), which may impair their ability to be at rest during quiet time with the dog, making the dog warier at these times. Other contexts seemed to provide important insights into when certain types of interaction might be perceived as aversive. For example, in relation to the child cuddling/kissing the dog, smaller dogs, older dogs and younger children were associated with higher levels of fearfulness. Whilst the latter effect may be explained by assuming that younger children may have less motor control and therefore show less refinement in their actions, previous research has also shown that smaller dogs show greater fear responses (65, 66), and that older dogs often show more fear in potentially stressful situations than younger dogs (67) with phobias increasing with age (68). In this case, it may be that smaller dogs are more vulnerable to being held too tightly and thus having a negative experience which increases over time.

As with Excitability scores, main caregivers tended to report lower signs of Fearfulness behavior than non-main caregivers of the dog across a range of situations perhaps indicating either a desensitization to the risk with repeat exposure (69), or reduced observation of the behavior (as a result of being more focused on the child) to recognize these behaviors, and/or their willingness to report them due to impression management as a “responsible dog owner.” Another possibility is that the difference lies with the non-caregiver, who could be more cautious as they are perhaps more naive of the situation (70). Determining which of these explanations is relevant may be important for developing rational child protection measures.

Fearfulness scores were important for predicting relationship satisfaction, with higher satisfaction associated with lower scores across a number of specific interactions. Higher scores across a range of child-dog interactions and in total also predicted greater health issues. These results not only highlight the relationship between fear/anxiety and stress-related illness (26), but might also suggest wider problems with the dog's behavior, since problematic behavior is a common reason for the bond to breakdown (25). The profile presented here, of dogs appearing anxious and having stress-related health issues would indicate that both medullary and cortical adrenal response are involved, i.e., that the dogs are suffering from stress associated with both frequent and prolonged exposure to situations they find aversive.

## Conclusion

The Lincoln Pet-dog Quality of Life (Lincoln P-QOL) scale is a relatively easy to use tool for dog caregivers to assess the impact of the child-dog relationship on the dog. The scale shows good validity in a range of child-dog interactions indicative of both immediate concerns and longer-term impacts relating to the well-being and quality of life of dogs. Our data highlight how interactions such as considerate child-dog physical affection and spending quiet time (e.g., reading) together may be beneficial to dogs, whereas other situations, beyond the more obvious ones like rough contact and child meltdown, such as grooming and bathing need careful monitoring. Fixed features relating to the dog, such as breed type and size seem to have little predictive value concerning their ability to adapt across contexts, i.e., there is no type of dog that is generally better with children. Further, our results indicate there is little value to be gained from distinguishing between families living with neuro-typically developing children and those with neuro-developmental disorders, when it comes to concerns about the well-being of the dog, and that each family should be evaluated for its own merits and challenges with respect to how the child interacts with the dog.

## Data Availability Statement

All datasets generated for this study are included in the manuscript/Supplementary Files.

## Ethics Statement

This studies involving human participants were reviewed and approved by University of Lincoln's College of Science Research Ethics Committee. The patients/participants provided their written informed consent to participate in this study.

## Author Contributions

SH and DM were responsible for study design, conception, and manuscript preparation. SH collected the data. BB assisted with data analysis, interpretation, and manuscript preparation.

### Conflict of Interest

The authors declare that the research was conducted in the absence of any commercial or financial relationships that could be construed as a potential conflict of interest.

## References

[1] Gadomski AM Scribani MB Krupa N Jenkins P Nagykaldi Z Olson AL Peer reviewed: pet dogs and children's health: opportunities for chronic disease prevention? Prev Chronic Dis. (2015) 12:E205 10.5888/pcd12.150204 26605705 PMC4674442

[2] Kerns KA Koehn AJ van Dulmen MH Stuart-Parrigon KL Coifman KG. Preadolescents' relationships with pet dogs: relationship continuity and associations with adjustment. Appl Dev Sci. (2017) 21:67–80. 10.1080/10888691.2016.1160781 29422765 PMC5800779

[3] Purewal R Christley R Kordas K Joinson C Meints K Gee N. Companion animals and child/adolescent development: a systematic review of the evidence. Int J Env Res Public Health. (2017) 14:234. 10.3390/ijerph14030234 28264460 PMC5369070

[4] Jalongo MR (editor). Introduction: children and the dogs in their lives. Children, Dogs and Education. New York, NY: Springer (2018). p. 1–18.

[5] Carlisle GK. The social skills and attachment to dogs of children with autism spectrum disorder. J Autism Dev Disord. (2015) 45:1137–45. 10.1007/s10803-014-2267-7 25308197

[6] Wright H Hall S Hames A Hardiman J Mills R Mills D. Acquiring a pet dog significantly reduces stress of primary carers for children with autism spectrum disorder: a prospective case control study. J Autism Dev Disord. (2015) 45:2531–40. 10.1007/s10803-015-2418-5 25832799 PMC4513200

[7] Wright H Hall S Hames A Hardiman J Mills R Team PP Pet dogs improve family functioning and reduce anxiety in children with autism spectrum disorder. Anthrozoös. (2015) 28:611–24. 10.1080/08927936.2015.1070003

[8] Hall SS Wright HF Hames A Mills DS The long-term benefits of dog ownership in families with children with autism. J Vet Behav Clin Appl Res. (2016) 13:46–54. 10.1016/j.jveb.2016.04.003

[9] Hall SS Wright HF Mills DS. What factors are associated with positive effects of dog ownership in families with children with autism spectrum disorder? The development of the Lincoln autism pet dog impact scale. PLoS ONE. (2016) 11:e0149736. 10.1371/journal.pone.0149736 26894820 PMC4760734

[10] Ward A Arola N Bohnert A Lieb R. Social-emotional adjustment and pet ownership among adolescents with autism spectrum disorder. J Commun Disord. (2017) 65:35–42. 10.1016/j.jcomdis.2017.01.002 28171740

[11] Silva K Lima M Santos-Magalhães A Fafiães C de Sousa L. Can dogs assist children with severe autism spectrum disorder in complying with challenging demands? An exploratory experiment with a live and a robotic dog. J Altern Complement Med. (2018) 24:238–42. 10.1089/acm.2017.0254 29116816

[12] Wright H Hall S Hames A Hardiman J Burgess A Mills R Effects of pet dogs for children with autism spectrum disorders (ASD) and their families: expectations versus reality. Hum Anim Interact Bull. (2016) 4:38–58.

[13] Powell L Chia D McGreevy P Podberscek AL Edwards KM Neilly B. Expectations for dog ownership: perceived physical, mental and psychosocial health consequences among prospective adopters. PLoS ONE. (2018) 13:e0200276. 10.1371/journal.pone.0200276 29979749 PMC6034856

[14] Hall SS Finka L Mills DS A systematic scoping review: what is the risk from child-dog interactions to dog quality of life? J Vet Behav. (2019) 33:16–26. 10.1016/j.jveb.2019.05.001

[15] Favrot C Linek M Mueller R Zini E International task force for canine atopic dermatitis development of a questionnaire to assess the impact of atopic dermatitis on health-related quality of life of affected dogs and their owners. Vet Dermatol. (2010) 21:63–9. 10.1111/j.1365-3164.2009.00781.x 20187912

[16] Iliopoulou MA Kitchell BE Yuzbasiyan-Gurkan V. Development of a survey instrument to assess health-related quality of life in small animal cancer patients treated with chemotherapy. J Am Vet Med Assoc. (2013) 242:1679–87. 10.2460/javma.242.12.1679 23725431

[17] Noli C Minafò G Galzerano M. Quality of life of dogs with skin diseases and their owners. Part 1: development and validation of a questionnaire. Vet Dermatol. (2011) 22:335–43. 10.1111/j.1365-3164.2010.00954.x 21410569

[18] Yeates J Main D. Assessment of companion animal quality of life in veterinary practice and research. J Small Anim Pract. (2009) 50:274–81. 10.1111/j.1748-5827.2009.00755.x 19527420

[19] Belshaw Z Asher L Harvey N Dean R. Quality of life assessment in domestic dogs: an evidence-based rapid review. Vet J. (2015) 206:203–12. 10.1016/j.tvjl.2015.07.016 26358965 PMC4641869

[20] Pirrone F Pierantoni L Mazzola SM Vigo D Albertini M Owner and animal factors predict the incidence of, and owner reaction toward, problematic behaviors in companion dogs. J Vet Behav Clin Appl Res. (2015) 10:295–301. 10.1016/j.jveb.2015.03.004

[21] Salman M New J John G Scarlett JM Kass PH Ruch-Gallie R. Human and animal factors related to relinquishment of dogs and cats in 12 selected animal shelters in the United States. J Appl Anim Welf Sci. (1998) 1:207–26. 10.1207/s15327604jaws0103_2 16363966

[22] Salman MD Hutchison J Ruch-Gallie R Kogan L New JC Jr Kass PH Behavioral reasons for relinquishment of dogs and cats to 12 shelters. J Appl Anim Welf Sci. (2000) 3:93–106. 10.1207/S15327604JAWS0302_2

[23] Arkow P. The humane society and the human-companion animal bond. Reflections on the broken bond. Vet Clin North Am Small Anim Pract. (1985) 15:455–66. 10.1016/S0195-5616(85)50317-4 3872525

[24] Marston LC Bennett PC Reforging the bond—towards successful canine adoption. Appl Anim Behav Sci. (2003) 83:227–45. 10.1016/S0168-1591(03)00135-7

[25] Diesel G Brodbelt D Pfeiffer DU. Characteristics of relinquished dogs and their owners at 14 rehoming centers in the United Kingdom. J Appl Anim Welf Sci. (2010) 13:15–30. 10.1080/10888700903369255 20017043

[26] Mills D Karagiannis C Zulch H Stress—its effects on health and behavior: a guide for practitioners. Vet Clin Small Anim Pract. (2014) 44:525–41. 10.1016/j.cvsm.2014.01.005 24766698

[27] Beerda B Schilder MB van Hooff JA de Vries HW Mol JA. Chronic stress in dogs subjected to social and spatial restriction. I. Behavioral responses. Physiol Behav. (1999) 66:233–42. 10.1016/S0031-9384(98)00289-3 10336149

[28] Luescher AU Reisner IR. Canine aggression toward familiar people: a new look at an old problem. Vet Clin North Am Small Anim Pract. (2008) 38:1107–30. 10.1016/j.cvsm.2008.04.008 18672156

[29] Mills D Westgarth C Dog Bites: a Multidisciplinary Perspective. Sheffield: 5M Publishing (2017).

[30] Melson GF Kahn PH Beck A Friedman B Roberts T Garrett E Children's behavior toward and understanding of robotic and living dogs. J Appl Dev Psychol. (2009) 30:92–102. 10.1016/j.appdev.2008.10.011

[31] Schalamon J Ainoedhofer H Singer G Petnehazy T Mayr J Kiss K. Analysis of dog bites in children who are younger than 17 years. Pediatrics. (2006) 117:e374–9. 10.1542/peds.2005-1451 16510617

[32] Reisner IR Nance ML Zeller JS Houseknecht EM Kassam-Adams N Wiebe DJ. Behavioural characteristics associated with dog bites to children presenting to an urban trauma centre. Inj Prev. (2011) 17:348–53. 10.1136/ip.2010.029868 21444335

[33] Handlin L Hydbring-Sandberg E Nilsson A Ejdebäck M Jansson A Uvnäs-Moberg K Short-term interaction between dogs and their owners: effects on oxytocin, cortisol, insulin and heart rate—an exploratory study. Anthrozoös. (2011) 24:301–15. 10.2752/175303711X13045914865385

[34] Petersson M Uvnäs-Moberg K Nilsson A Gustafson L-L Hydbring-Sandberg E Handlin L. Oxytocin and cortisol levels in dog owners and their dogs are associated with behavioral patterns: an exploratory study. Front Psychol. (2017) 8:1796. 10.3389/fpsyg.2017.01796 29081760 PMC5645535

[35] Handlin L Nilsson A Ejdebäck M Hydbring-Sandberg E Uvnäs-Moberg K Associations between the psychological characteristics of the human–dog relationship and oxytocin and cortisol levels. Anthrozoös. (2012) 25:215–28. 10.2752/175303712X13316289505468

[36] Burrows KE Adams CL Millman ST. Factors affecting behavior and welfare of service dogs for children with autism spectrum disorder. J Appl Anim Welf Sci. (2008) 11:42–62. 10.1080/10888700701555550 18444026

[37] Marinelli L Normando S Siliprandi C Salvadoretti M Mongillo P. Dog assisted interventions in a specialized centre and potential concerns for animal welfare. Vet Res Commun. (2009) 33:93–5. 10.1007/s11259-009-9256-x 19578960

[38] Hall SS Wright HF Mills DS. Parent perceptions of the quality of life of pet dogs living with neuro-typically developing and neuro-atypically developing children: an exploratory study. PLoS ONE. (2017) 12:e0185300. 10.1371/journal.pone.0185300 28953961 PMC5617192

[39] Palestrini C Calcaterra V Cannas S Talamonti Z Papotti F Buttram D Stress level evaluation in a dog during animal-assisted therapy in pediatric surgery. J Vet Behav Clin Appl Res. (2017) 17:S221–2. 10.1016/j.jveb.2016.09.003

[40] McCullough A Jenkins MA Ruehrdanz A Gilmer MJ Olson J Pawar A Physiological and behavioral effects of animal-assisted interventions on therapy dogs in pediatric oncology settings. Appl Anim Behav Sci. (2018) 200:86–95. 10.1016/j.applanim.2017.11.014

[41] Taylor KD Mills DS The development and assessment of temperament tests for adult companion dogs. J Vet Behav Clin Appl Res. (2006) 1:94–108. 10.1016/j.jveb.2006.09.002

[42] Kiddie JL Collins LM Development and validation of a quality of life assessment tool for use in kennelled dogs (*Canis familiaris*). Appl Anim Behav Sci. (2014) 158:57–68. 10.1016/j.applanim.2014.05.008

[43] Nestle Purina Purina Dog Condition Chart. (2014). Available online at: https://oregonvma.org/files/Purina-Dog-Condition-Chart.pdf (accessed June 17, 2019).

[44] Hendrick SS A generic measure of relationship satisfaction. J Marriage Fam. (1988) 50:93–9. 10.2307/352430

[45] Cronbach LJ Coefficient alpha and the internal structure of tests. Psychometrika. (1951) 16:297–334. 10.1007/BF02310555

[46] Hendrick SS Dicke A Hendrick C The relationship assessment scale. J Soc Pers Relat. (1998) 15:137–42. 10.1177/0265407598151009

[47] Perneger TV. What's wrong with Bonferroni adjustments. BMJ. (1998) 316:1236–8. 10.1136/bmj.316.7139.1236 9553006 PMC1112991

[48] Clark LA Watson D Constructing validity: basic issues in objective scale development. Psychol Assess. (1995) 7:309 10.1037/1040-3590.7.3.309 PMC6754793 30896212

[49] Casey RA Loftus B Bolster C Richards GJ Blackwell EJ Human directed aggression in domestic dogs (*Canis familiaris*): occurrence in different contexts and risk factors. Appl Anim Behav Sci. (2014) 152:52–63. 10.1016/j.applanim.2013.12.003

[50] Mariti C Falaschi C Zilocchi M Fatjó J Sighieri C Ogi A Analysis of the intraspecific visual communication in the domestic dog (*Canis familiaris*): a pilot study on the case of calming signals. J Vet Behav. (2017) 18:49–55. 10.1016/j.jveb.2016.12.009

[51] Kerswell KJ Bennett PJ Butler KL Hemsworth PH Self-reported comprehension ratings of dog behavior by puppy owners. Anthrozoös. (2009) 22:183–93. 10.2752/175303709X434202

[52] Tami G Gallagher A Description of the behaviour of domestic dog (*Canis familiaris*) by experienced and inexperienced people. Appl Anim Behav Sci. (2009) 120:159–69. 10.1016/j.applanim.2009.06.009

[53] Mariti C Gazzano A Moore JL Baragli P Chelli L Sighieri C Perception of dogs' stress by their owners. J Vet Behav Clin Appl Res. (2012) 7:213–9. 10.1016/j.jveb.2011.09.004

[54] Lakestani NN Donaldson ML Waran N Interpretation of dog behavior by children and young adults. Anthrozoös. (2014) 27:65–80. 10.2752/175303714X13837396326413

[55] Schmitz C Martineau J Barthélémy C Assaiante C. Motor control and children with autism: deficit of anticipatory function? Neurosci Lett. (2003) 348:17–20. 10.1016/S0304-3940(03)00644-X 12893415

[56] Dewey D Cantell M Crawford SG Motor and gestural performance in children with autism spectrum disorders, developmental coordination disorder, and/or attention deficit hyperactivity disorder. J Int Neuropsychol Soc. (2007) 13:246–56. 10.1017/S1355617707070270 17286882

[57] Tomchek SD Dunn W. Sensory processing in children with and without autism: a comparative study using the short sensory profile. Am J Occup Ther. (2007) 61:190–200. 10.5014/ajot.61.2.190 17436841

[58] Mazefsky CA Herrington J Siegel M Scarpa A Maddox BB Scahill L. The role of emotion regulation in autism spectrum disorder. J Am Acad Child Adolesc Psychiatry. (2013) 52:679–88. 10.1016/j.jaac.2013.05.006 23800481 PMC3719386

[59] Curb LA Abramson CI Grice JW Kennison SM The relationship between personality match and pet satisfaction among dog owners. Anthrozoös. (2013) 26:395–404. 10.2752/175303713X13697429463673

[60] Baenninger R. On yawning and its functions. Psychon Bull Rev. (1997) 4:198–207. 10.3758/BF03209394 21331826

[61] Beerda B Schilder M Van Hooff J De Vries H Mol J Behavioural and hormonal indicators of enduring environmental stress in dogs. Anim Welf Potters Bar. (2000) 9:49–62. Available online at: https://www.cabdirect.org/cabdirect/abstract/20002209235

[62] Mills DS Perspectives on assessing the emotional behavior of animals with behavior problems. Curr Opin Behav Sci. (2017) 16:66–72. 10.1016/j.cobeha.2017.04.002

[63] Lacreuse A Parr L Chennareddi L Herndon JG. Age-related decline in cognitive flexibility in female chimpanzees. Neurobiol Aging. (2018) 72:83–8. 10.1016/j.neurobiolaging.2018.08.018 30237074 PMC6215734

[64] Corbett BA Constantine LJ Hendren R Rocke D Ozonoff S. Examining executive functioning in children with autism spectrum disorder, attention deficit hyperactivity disorder and typical development. Psychiatry Res. (2009) 166:210–22. 10.1016/j.psychres.2008.02.005 19285351 PMC2683039

[65] Uhde TW Malloy LC Slate SO. Fearful behavior, body size, and serum IGF-I levels in nervous and normal pointer dogs. Pharmacol Biochem Behav. (1992) 43:263–9. 10.1016/0091-3057(92)90667-5 1409812

[66] Arhant C Bubna-Littitz H Bartels A Futschik A Troxler J Behaviour of smaller and larger dogs: effects of training methods, inconsistency of owner behaviour and level of engagement in activities with the dog. Appl Anim Behav Sci. (2010) 123:131–42. 10.1016/j.applanim.2010.01.003

[67] Döring D Roscher A Scheipl F Küchenhoff H Erhard MH. Fear-related behaviour of dogs in veterinary practice. Vet J. (2009) 182:38–43. 10.1016/j.tvjl.2008.05.006 18700181

[68] Bamberger M Houpt KA. Signalment factors, comorbidity, and trends in behavior diagnoses in dogs: 1,644 cases (1991–2001). J Am Vet Med Assoc. (2006) 229:1591–601. 10.2460/javma.229.10.1591 17107314

[69] Mrug S Madan A Windle M. Emotional desensitization to violence contributes to adolescents' violent behavior. J Abnorm Child Psychol. (2016) 44:75–86. 10.1007/s10802-015-9986-x 25684447 PMC4539292

[70] Song H Schwarz N If it's difficult to pronounce, it must be risky: fluency, familiarity, and risk perception. Psychol Sci. (2009) 20:135–8. 10.1111/j.1467-9280.2009.02267.x 19170941

[71] Hoffman CL Chen P Serpell JA Jacobson KC. Do dog behavioral characteristics predict the quality of the relationship between dogs and their owners? Hum Anim Interact Bull. (2013) 1:20. 25685855 PMC4326091

