# Supplementary material for: Developing and Assessing the Validity of a Scale to Assess Pet Dog Quality of Life: Lincoln P-QoL
Source: Front Vet Sci. 2019 Sep 26;6:326. doi: 10.3389/fvets.2019.00326 (PMC6775215; doi:10.3389/fvets.2019.00326)
Supplement: Supplementary file 1 [file Table_1.DOCX]

Supplementary Table 1. *P* values, to accompany Table 6 and results detailing the effect of child-dog interactions on pet dog quality of life score constructs.

| **Comparison between Interactions** | **Excitement** | **Calmness** | **Fearfulness** |
| --- | --- | --- | --- |
|  | *p values* | | |
| Meltdown-Car | 0.000 | 0.000 | 0.000 |
| Meltdown-Child visitors | 0.000 | 0.003 | 0.000 |
| Meltdown-Cuddle/Kiss | 0.000 | 0.000 | 0.000 |
| Meltdown- Groom/Bathe | 0.667 | 0.000 | 0.113 |
| Meltdown- High energy activities | 0.000 | 0.000 | 0.000 |
| Meltdown- Loud toys | 0.000 | 0.000 | 0.000 |
| Meltdown- Disrupt routine | 0.652 | 0.000 | 0.000 |
| Meltdown- Rough contact | 0.009 | 0.000 | 0.001 |
| Meltdown – Quiet time | 0.035 | 0.000 | 0.000 |
| Meltdown- Disturb safe place | 0.122 | 0.000 | 0.000 |
| Car- Child visitors | 0.000 | 0.049 | 0.003 |
| Car- Cuddle/Kiss | 0.000 | 0.000 | 0.342 |
| Car-Groom/Bathe | 0.000 | 0.070 | 0.015 |
| Car-High energy activities | 0.000 | 0.000 | 0.000 |
| Car- Loud toys | 0.000 | 0.002 | 0.733 |
| Car- Disrupt routine | 0.000 | 0.000 | 0.258 |
| Car- Rough contact | 0.000 | 0.000 | 0.000 |
| Car- Quiet time | 0.000 | 0.000 | 0.000 |
| Car- Disturb safe place | 0.000 | 0.645 | 0.685 |
| Child visitors- Cuddle/Kiss | 0.000 | 0.000 | 0.036 |
| Child visitors- Groom/Bathe | 0.000 | 0.002 | 0.000 |
| Child visitors- High energy activities | 0.000 | 0.000 | 0.000 |
| Child visitors- Loud toys | 0.000 | 0.000 | 0.079 |
| Child visitors- Disrupt routine | 0.000 | 0.000 | 0.845 |
| Child visitors- Rough contact | 0.000 | 0.000 | 0.000 |
| Child visitors- Quiet time | 0.000 | 0.000 | 0.000 |
| Child visitors- Disturb safe place | 0.000 | 0.012 | 0.058 |
| Cuddle/Kiss- Groom/Bathe | 0.000 | 0.000 | 0.001 |
| Cuddle/Kiss- High energy activities | 0.000 | 0.000 | 0.000 |
| Cuddle/Kiss- Loud toys | 0.425 | 0.000 | 0.739 |
| Cuddle/Kiss- Disrupt routine | 0.000 | 0.000 | 0.055 |
| Cuddle/Kiss- Rough contact | 0.000 | 0.000 | 0.000 |
| Cuddle/Kiss- Quiet time | 0.000 | 0.000 | 0.000 |
| Cuddle/Kiss- Disturb safe place | 0.000 | 0.000 | 0.383 |
| Groom/Bathe- High energy activities | 0.000 | 0.075 | 0.000 |
| Groom/Bathe- Loud toys | 0.002 | 0.055 | 0.158 |
| Groom/Bathe- Disrupt routine | 0.412 | 0.011 | 0.002 |
| Groom/Bathe-Rough contact | 0.066 | 0.000 | 0.031 |
| Groom/Bathe- Quiet time | 0.298 | 0.000 | 0.000 |
| Groom/Bathe- Disturb safe place | 0.707 | 0.390 | 0.011 |
| High energy activities- Loud toys | 0.000 | 0.252 | 0.000 |
| High energy activities- Disrupt routine | 0.000 | 0.097 | 0.000 |
| High energy activities- Rough contact | 0.000 | 0.000 | 0.000 |
| High energy activities- Quiet time | 0.000 | 0.000 | 0.699 |
| High energy activities- Disturb safe place | 0.000 | 0.514 | 0.000 |
| Loud toys- Routine | 0.001 | 0.034 | 0.290 |
| Loud toys- Rough contact | 0.001 | 0.018 | 0.000 |
| Loud toys- Quiet time | 0.000 | 0.000 | 0.000 |
| Loud toys- Disturb safe place | 0.001 | 0.069 | 0.233 |
| Routine- Rough contact | 0.455 | 0.359 | 0.000 |
| Routine- Quiet time | 0.018 | 0.000 | 0.000 |
| Routine- Disturb safe place | 0.272 | 0.002 | 0.005 |
| Rough contact- Quiet time | 0.875 | 0.000 | 0.000 |
| Rough contact- Disturb safe place | 0.573 | 0.000 | 0.000 |
| Quiet time- Disturb safe place | 0.313 | 0.000 | 0.000 |
